# Seroprevalence of Rift Valley fever virus in domestic ruminants of various origins in two markets of Yaoundé, Cameroon

**DOI:** 10.1371/journal.pntd.0010683

**Published:** 2022-08-11

**Authors:** Francine Yousseu Sado, Huguette Simo Tchetgna, Basile Kamgang, Doumani Djonabaye, Emmanuel Nakouné, Philip J. McCall, Roland Ndip Ndip, Charles S. Wondji

**Affiliations:** 1 Microbiology and Parasitology Department, Centre for Research in Infectious Diseases, Yaounde, Cameroon; 2 Department of Microbiology and Parasitology, Faculty of Science, University of Buea, Cameroon; 3 Department of Biochemistry, Laboratory of Pharmacology and Toxicology, University of Yaounde I, Cameroon; 4 Laboratory of Influenza, viral hemorrhagic fever, arbovirus, zoonosis, emerging and re-emerging viruses, Institut Pasteur of Bangui, Central African Republic; 5 Vector Biology Department, Liverpool School of Tropical Medicine, United Kingdom; NIAID Integrated Research Facility, UNITED STATES

## Abstract

**Background:**

Rift Valley fever (RVF) is a mosquito-borne zoonosis endemic in Africa. With little known of the burden or epidemiology of RVF virus (RVFV) in Cameroon, this study aimed to determine the seroprevalence of RVFV in domestic ruminants of various origins in two markets of Yaoundé, Cameroon.

**Methodology/Principal findings:**

The origin of animals randomly sampled at two livestock markets in Yaoundé were recorded and plasma samples collected for competitive and capture Enzyme-linked Immunosorbent Assay (ELISA) to determine the prevalence of Immunoglobulins G (IgG) and Immunoglobulins M (IgM) antibodies. Following ELISA IgM results, a real-time reverse transcription-polymerase chain reaction (qRT-PCR) was performed to detect RVFV RNA. In June-August 2019, February-March 2020, and March-April 2021, 756 plasma samples were collected from 441 cattle, 168 goats, and 147 sheep. RVFV IgG seroprevalence was 25.7% for all animals, 42.2% in cattle, 2.7% in sheep, and 2.4% in goats. However, IgM seroprevalence was low, at 0.9% in all animals, 1.1% in cattle, 1.4% in sheep, and 0% in goats. The seroprevalence rates varied according to the animal’s origin with the highest rate (52.6%) in cattle from Sudan. In Cameroon, IgG and IgM rates respectively were 45.1% and 2.8% in the North, 44.8% and 0% in the Adamawa, 38.6% and 1.7% in the Far-North. All IgM positive samples were from Cameroon. In cattle, 2/5 IgM positive samples were also IgG positive, but both IgM positive samples in sheep were IgG negative. Three (42.9%) IgM positive samples were positive for viral RVFV RNA using qRT-PCR but given the high ct values, no amplicon was obtained.

**Conclusion/Significance:**

These findings confirm the circulation of RVFV in livestock in Cameroon with prevalence rates varying by location. Despite low IgM seroprevalence rates, RVF outbreaks can occur without being noticed. Further epidemiological studies are needed to have a broad understanding of RVFV transmission in Cameroon.

**Summary**

Little is known about the epidemiology of Rift Valley fever (RVF) in Cameroon. Thus, this study aimed to determine the seroprevalence of RVF virus (RVFV) in domestic ruminants of various origins at two markets in Yaoundé, Cameroon. A questionnaire was used to assess animal origins and plasma samples were collected for competitive and capture Enzyme-linked Immunosorbent Assay (ELISA) to determine the prevalence of Immunoglobulins G (IgG) and Immunoglobulins M (IgM) antibodies. Then, a real-time reverse transcription-polymerase chain reaction (qRT-PCR) was performed to detect the viral RNA in anti-RVFV IgM positive samples. Overall, 756 samples were collected in 2019, 2020, and 2021 from 441 cattle, 168 goats, and 147 sheep. RVFV IgG seroprevalence was 25.7% for all animals, but higher in cattle (42.2%) than sheep (2.7%), and goats (2.4%). However, IgM seroprevalence was low in all animals (0.9%). The IgM seroprevalence in sheep, cattle, and goats was 1.4%, 1.1%, and 0% respectively. RVFV seroprevalence was higher in livestock from the North region of Cameroon. Three IgM positive samples were positive by qRT-PCR, however, no amplicon was obtained.

## Background

Rift Valley fever (RVF) is an important emerging, mosquito-borne zoonotic disease that is responsible of periodic outbreaks in ruminants and humans [1]. The disease is caused by a single-stranded, segmented RNA virus belonging to the genus *Phlebovirus* within the family *Phenuiviridae* and the order *Bunyavirales* [2]. While mild infection in wild and domestic ungulates may be characterized by symptoms such as fever and weakness, the severe forms lead to necrotic hepatitis, abortions near 100% in livestock, and a hemorrhagic state leading to high mortality, particularly among newborns [3]. In humans, 50 to 95% of individuals infected with RVF virus (RVFV) present with a flu-like illness, with a reported overall case fatality rate of 1–3% which can be as high as 34% in naïve population [4–6]. Actually, 2% of RVFV infections can evolve into hemorrhagic complications, hepatitis, neurological disorders, and blindness [3,7], leading to death in almost 50% of those cases [8].

RVFV is transmitted between vertebrate animals by the bite of several mosquito genera, mainly *Aedes* and *Culex* whereas transmission to humans can also occur through direct contact with tissues and body fluids of infected animals or consumption of infected raw milk [6,9,10]. RVFV persists between seasons and epidemics via a vertical transmission with desiccation-resistant *Aedes* mosquito eggs that are viable for several months [11,12]. Epizootics of RVF are sporadic and are often linked to persistent heavy rainfall associated with dam construction in West Africa (i.e. the Senegal river basin dam) and La Nina events in East Africa and changes in the habitat of RVFV vectors [1,13,14]. The virus transmission is thereafter amplified by various species of mosquito including *Aedes* spp., *Culex* spp., and *Anopheles* spp. infected mosquitoes when flooding occurs [8,15]. Control of RVF relies on surveillance, early detection, laboratory confirmation, and application of zoo-sanitary measures [16]. In endemic countries, the vaccine is often used to prevent the disease in livestock but it is not yet the case for humans [17]. Hence, the surveillance of this disease remains the key point for outbreak prevention.

RVF was first described in Cameroon in 1967 from sheep and wild animals (gazelle, buffalo) in the North region [18]. Unlike neighboring countries that have recently reported RVF outbreaks, Cameroon has not yet experienced any documented epidemic of the disease. There is limited data on the epidemiology of RVF in Cameroon [19–22], with the lack of a surveillance system. As a step toward routine monitoring of the enzootic circulation of this disease in Cameroon, to prevent potential outbreaks as seen in neighboring countries, this study aimed to determine the seroprevalence of RVFV in cattle, sheep, and goats from different origins in Cameroon, sold in markets of Yaoundé, the capital city of the country.

## Materials and methods

### Ethics statement

The study protocol was implemented with approval from the Regional Delegation of the Ministry of Livestock, Fisheries, and Animal Industries (MINEPIA), Authorization N°000151/L/MINEPIA/SG/DREPIA-CE/SRDPIA and 00034/L/MINEPIA/SG/DREPIA-CE. Oral consent for blood sampling of herds was obtained from owners.

### Description of the study sites

**Fig 1:**
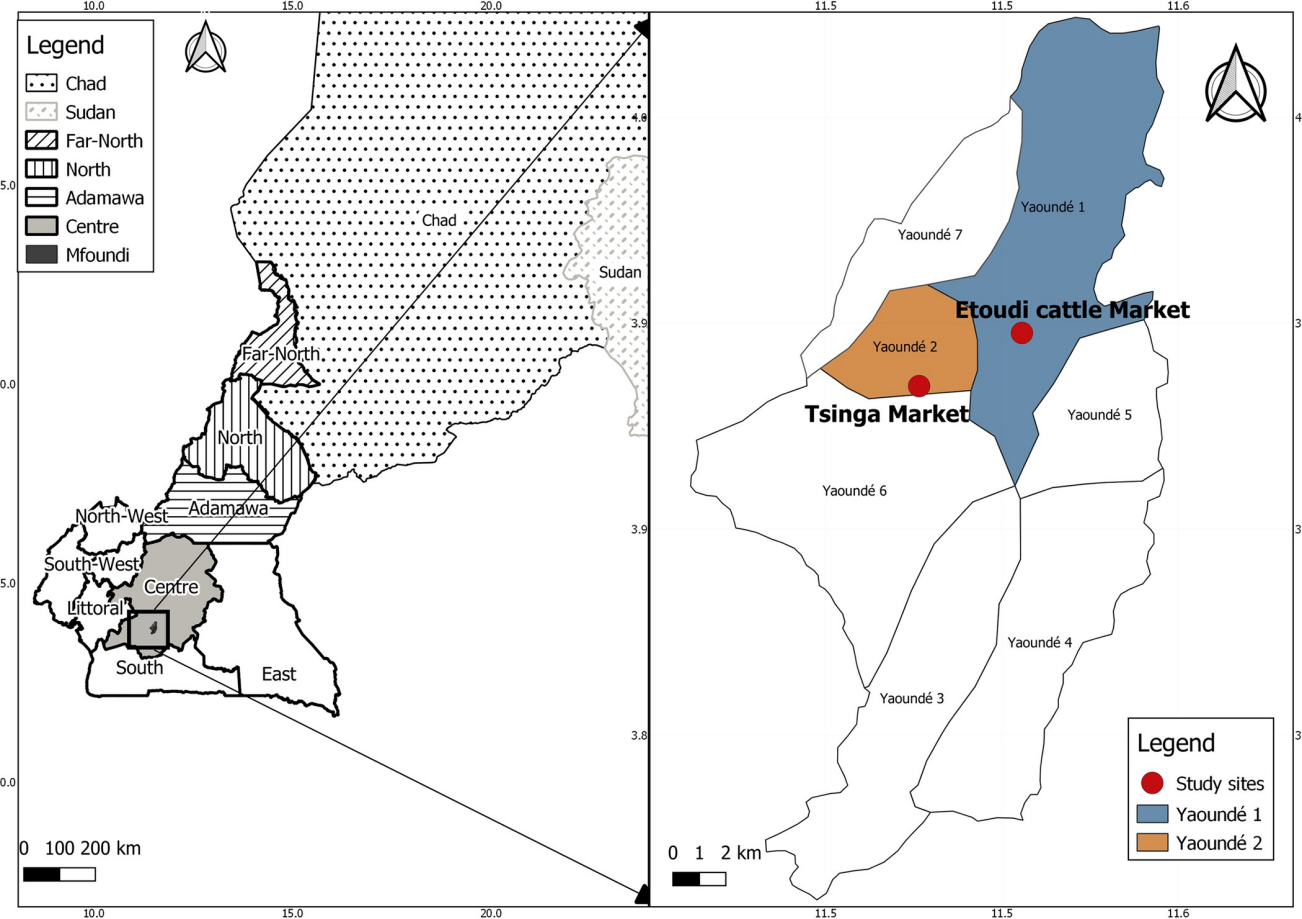
Geographic repartition of the animal used for the study. Panel A represents the locations and origins of the animals. The northern region where animals originated from Cameroon is represented by the black stripes, oblic stripes represent the Far-North, vertical, the North, and horizontal stripes the Adamawa region. Neighboring countries are represented by black dots (Chad) and dash grey (Sudan). Light grey represents the Centre region with the Mfoundi division in dark grey. Panel B presents the markets from where the cattle (in dark blue) and the small ruminants (in orange) have been sampled. QGIS version 3.14.16, was used to generate the map using open access sharefiles (https://gadm.org/)

The study was carried out in the main livestock markets of Yaoundé, Mfoundi Division, Centre region of Cameroon namely Etoudi (3°55’N, 11°31’36” E) in Yaoundé 1 district for cattle, and Tsinga market (3°53’55” N, 11°29’30” E) in Yaoundé 2 district for goats and sheep (Fig 1). The cattle market of Etoudi is a periodic market where about 1500 to 2000 cattle are traded each market day while the small ruminant market at Tsinga holds about 1000 goats and 1000 sheep weekly. In Etoudi market, the cattle are brought and sold three times a week (Wednesday, Friday, and Sunday) while in Tsinga, small ruminants arrived twice a week (Tuesday and Thursday).

### Sample collection and processing

Our study was carried out in two periodic markets. On the market day, our objective was to sample all the available herds. Consent was obtained from the owners of each herd, a questionnaire was administered to determine the size of the herd, the sex, and origin, then we randomly sampled 10% of the herd according to the herd size. Additionally, animals’ ages were determined using their horns and dentition. Sampling was carried out during the dry season, from July to August 2019 and February to March 2020, and during the rainy season, in June 2019, then from March to April 2021, in those two markets [23]. Blood was collected from the jugular vein of cattle, goats, and sheep into EDTA tubes and preserved in a cooler for transportation to the laboratory. Plasma was obtained after centrifugation at 2500 rpm for 10 min, then transferred into cryotubes and stored at -20°C until analysis.

### Anti-RVFV IgG antibody screening

A Competitive Enzyme-Linked Immunosorbent Assay (c-ELISA) for the detection of IgG antibodies directed against the nucleoprotein (NP) of RVFV was performed according to the manufacturer’s instructions using 50 μL of plasma (Innovative Diagnostics, Grabels, France). The absorbance was measured at 450nm using the Biochrom EZ Read 400 ELISA Microplate Reader with Galapagos for EZ Read Software (ThermoScientific, Cambridge, Cambridgeshire, United Kingdom). The test was validated when the mean value of the negative control optical density (OD) (ODnc) was greater than 0.7 (ODnc > 0.7) and when the mean value of the positive control OD (OD_PC_) was less than 30% of the ODnc (OD_PC_/ ODnc < 0.3).

Then, the inhibition rate was calculated according to the following formula:

$$\frac{S}{N}(\%)=\frac{ODs}{ODnc}x100$$

With OD: optical density; nc: negative control; S: sample; S/N: competition percentage. S/N values lower than or equal to 40% were considered positive, values above 50% negative, and values in between inconclusive.

### Anti-RVFV IgM antibody screening

Additionally to anti-RVFV IgG detection, all the samples were analyzed using the IgM capture ELISA to specifically detect anti-RVFV IgM antibodies according to the manufacturer’s instructions (Innovative Diagnostics, Grabels, France). 10 μL of test samples and controls were added to the even and odd microwells respectively for the analysis. After the obtention of the optical density (OD) at 450 nm, the net sample OD (Net OD = OD_even well_−OD_odd well_), was determined. The test was validated when the mean value of the net positive control OD (net OD_PC_) was greater than 0.4 (net OD_PC_ > 0.4) and the ratio of the mean values of the net positive (net OD_PC_) and negative control OD (net OD_NC_) was greater than 3 (net OD_PC /_ net OD_NC_ >3).

Then the formula below was used to calculate the positivity percentage (S/P %);

$$\frac{S}{P}(\%)=\frac{netODs}{netODpc}x100$$

With OD: optical density, pc: positive control, S: sample, S/P: positivity percentage

S/P values above 50% were considered positive, values lower than or equal to 40%, negative, and values in between inconclusive.

### Detection of the RVFV and *Phlebovirus* RNA in anti-RVFV IgM positive samples

Viral RNA was extracted from anti-RVFV IgM positive plasma samples using the QIAamp Viral RNA Mini Kit (Qiagen, Hilden, Germany) according to the manufacturer’s instructions. Then, the cDNA was synthesized using the High-capacity cDNA reverse transcription kit (Applied Biosystems, Foster City, California, USA). Real-time RT-PCR (qRT-PCR) was carried out using 5 μL of cDNA and the TaqMan Universal real-time PCR Master Mix kit (Applied Biosystems, Foster City, California, USA) with 400 nM of each primer as described elsewhere [24] targeting a portion of the S genomic segment. PCR amplification was completed using a Stratagene Mx3005P real-time polymerase chain reaction (qPCR) system (Agilent Technologies, Santa Clara, California, USA). Furthermore, the qRT-PCR positive samples were further analyzed by conventional RT-PCR using primers targeting a fragment of the L segment of *Phlebovirus* as described elsewhere [25]. Briefly, 5 μl of the cDNA was added to 10 μl reaction mixture containing 1.5 μl of Taq Buffer B (1X), 1.5 mM of MgCl_2_, 25mM dNTP Mix, and 0.4μM of each primer using the KapaTaq PCR kit (Kapa Biosystems, Wilmington, Massachusetts, USA). Reactions were subjected to an initial cycle of 95°C for 5 min followed by 45 PCR cycles at 94°C for 30 s, 58°C for 1 min, and 72°C for 1 min, then the final extension was done at 72°C for 10 min. A nested PCR reaction mixture, containing the same concentration as stated above with a second set of primers was then performed at a 50°C annealing temperature. RT-PCR products were visualized on a 2% agarose gel using the SYBR Safe DNA Gel Stain (Invitrogen, Massachusetts, USA) and a 100 bp Hyper Ladder molecular weight marker (Meridian Bioscience, Bioline, Cincinnati, Ohio, USA) for an amplicon of 438bp.

### Data analysis

The serological data were analyzed using R software version 4.0.3 for windows via RStudio Version 1.3.1093 [26]. Overall, seroprevalence with 95% confidence intervals was calculated for the study site and origins of cattle. Seroprevalence rates were compared between origins and seasons using the Fisher exact test. The outcome variables were IgG, and IgM positivity and independent variables were the origin of livestock, animal species, and seasons. Subsequently, cross-tabulation and Chi-Square or Fisher exact test were used to examine the relationships between variables. QGIS version 3.14.16, was used to generate the map using open access sharefiles (https://gadm.org/). Figures were generated using GraphPad Prism version 8.0.0 for Windows (GraphPad Software, San Diego, California, USA, “www.graphpad.com”).

## Results

### Origin of the animals sampled and IgG and IgM seroprevalence rates

**Fig 2:**
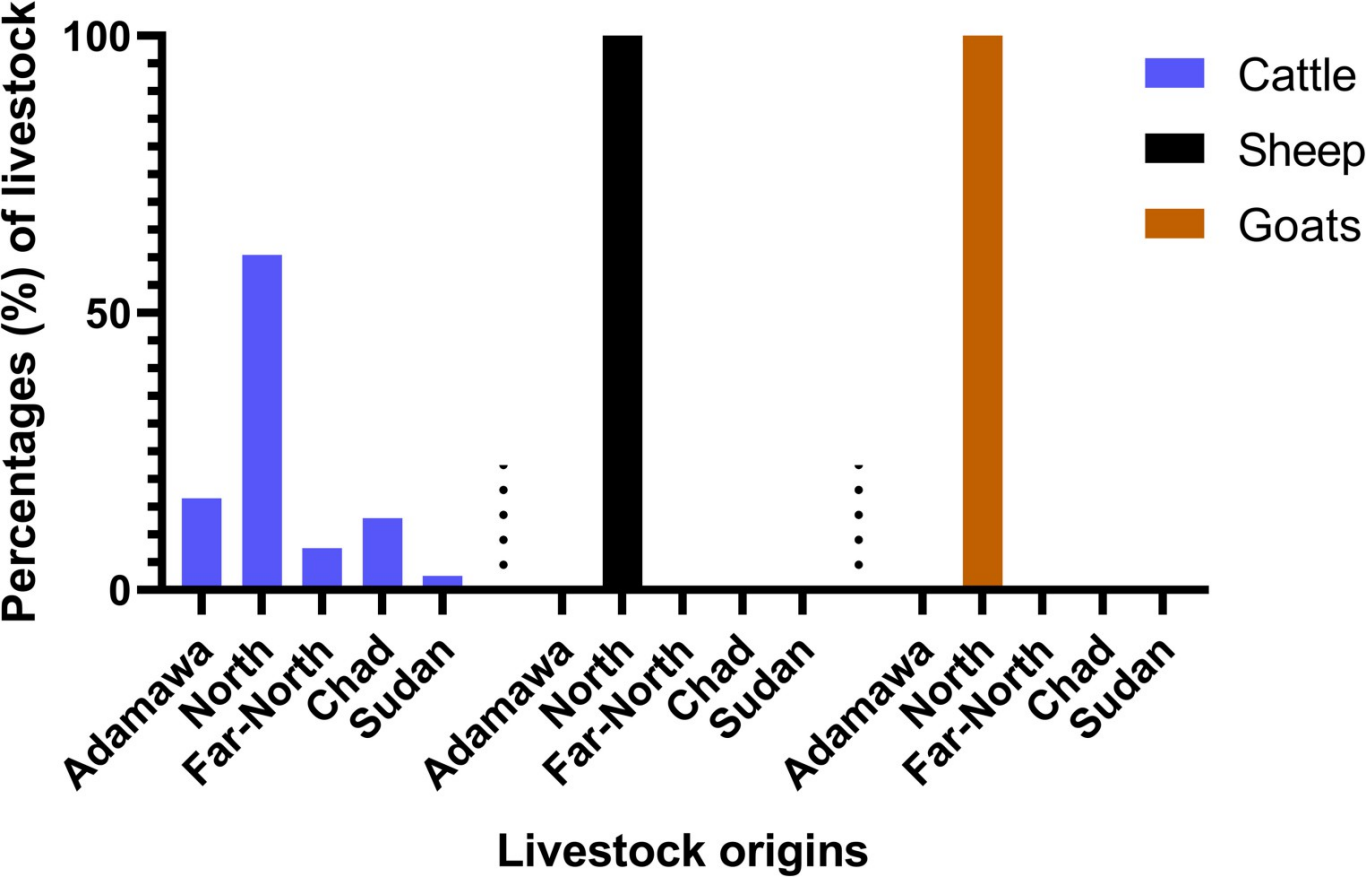
Distribution of animal species per origin. Three species of animal were sampled: cattle (blue), sheep (black), and goats (orange). Cattle originated from three different countries, Cameroon (Adamawa, North and Far-North regions), Chad, and Sudan whereas sheep and goats originated from the North region of Cameroon.

Overall, 756 adult animals (692 males and 64 females) were sampled, comprising 441 cattle (438 males, 3 females), 168 goats (119 males, 49 females), and 147 sheep (135 males, 12 females). Cattle were mostly from Cameroon (324/441; 73.5%), Chad (98/441; 22.2%), and Sudan (19/441; 4.3%). However, all sheep and goats only came from the North region of Cameroon. Among the cattle from Cameroon, 43.8% (142/324) were from the North region, 38.6% (125/324) from the Adamawa, and 17.6% (57/324) from the Far-North (Fig 2).

**Fig 3:**
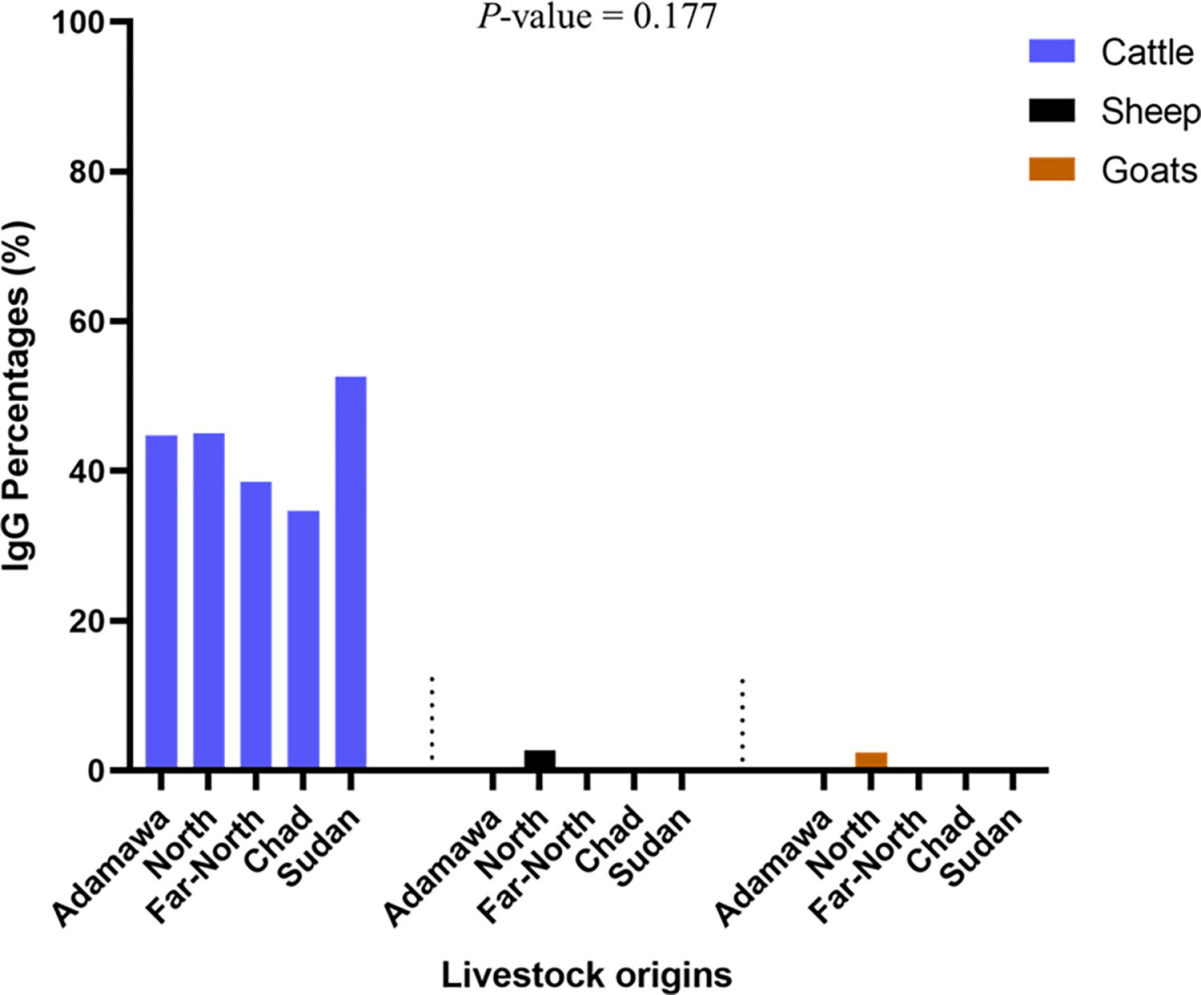
Anti-RVFV IgG seroprevalence distribution per species and origins. Anti-RVFV IgG positive samples from cattle are represented in blue whereas sheep are represented in black and Goats in orange.

Seroprevalence of anti-RVFV IgG antibodies in all animals was 25.7% (194/756, 95% CI: [22.6–28.9]) with the highest IgG seroprevalence in cattle, 42.2% (186/441, 95% CI: [37.5–46.9]), while sheep and goats both reported low IgG seroprevalences of 2.7% (4/147, 95% CI: [0.7–6.8]) and 2.4% (4/168, 95% CI: [0.7–5,9]), respectively (Fig 3). Furthermore, we observed 1.1% (5/441), 2.4% (4/168), and 1.4% (2/147) inconclusive results for IgG detection in cattle, sheep, and goats, respectively. However, these results were excluded from subsequent analysis. Statistical analysis revealed a significant difference in IgG seroprevalence in cattle compared to small ruminants (χ^2^ = 151, *P*-value < 0.0001). Cattle from Sudan had the highest IgG seroprevalence (10/19, 52.6%), followed by those from Cameroon (142/324, 43.8%), with the North region baring the highest IgG (64/142, 45.1%) seroprevalence. We also identified 34.7% (34/98) IgG seroprevalence in cattle from Chad. The difference in IgG seroprevalence observed between countries was not statistically significant (X^2^ = 3.5, *P*-value = 0.177).

Anti-RVFV IgM antibodies seroprevalence was 0.9% (7/756, 95% CI: [0.4–1.9]) in all the animals. The IgM seroprevalence was 1.1% (5/441, 95% CI: [0.4–2.6]) in cattle and 1.4% (2/147, 95% CI: [0.2–4.8]) in sheep. However, no IgM was detected in samples from goats and no inconclusive result was obtained. All anti-RVFV IgM positive samples in cattle were from the North (four cattle) and Far North (one cattle) regions of Cameroon. Additionally, 2 out of 5 IgM-positive samples in cattle were also IgG-positive. Furthermore, the two IgM-positive sheep did not have any anti-RVFV IgG antibodies. There were no species differences in IgM seroprevalence (*P*-value = 0.705).

qRT-PCR allowed the detection of 3 RVFV positive samples on 7 anti-RVFV IgM positive samples obtained (3/7. 42.9%, 95% CI: [9.9–81.6]), with 2 from cattle, one of which was anti-RVFV IgG positive and another IgG inconclusive and one from sheep with cycle threshold values (Ct) of 39, 39 and 40 respectively. These qRT-PCR positive samples were from the North region of Cameroon. No amplicon was obtained through the amplification of the L Segment of *Phlebovirus*.

### Seasonal variation of the seroprevalence of IgG and IgM anti-RVFV antibodies

A seasonal variation in the seroprevalence of IgG and IgM anti RVFV antibodies was observed in cattle. Indeed, a higher seroprevalence of IgG (37%) was recorded during the dry season. An opposite observation was made for goats where the highest IgG prevalence (0.7%) was obtained during the rainy season. No seasonal variation in IgG seroprevalence was observed for sheep (Table 1). The seasonal difference in the IgG seroprevalence in cattle was statistically significant (X^2^:135.45, *P-*value < 0.0002) (Table 1).

**Table 1:** Seasonal distribution of RVF seroprevalence in livestock.

Seasons	Cattle		Goats		Sheep			Total
	IgG P/T (%)	IgM P/T (%)	IgG P/T (%)	IgM P/T (%)	IgG P/T (%)	IgM P/T (%)	RT-PCR P/T (%)	
Rainy season	75/456 (16.5)	3/456 (0.7)	3/456 (0.7)	0	2/456 (0.4)	2/456 (0.4)	2/5 (40)	456 (60.3)
Dry season	111/300 (37)	2/ 300 (0.7)	1/300 (0.3)	0	2/300 (0.7)	0	1/2 (50)	300 (39.7)
*P*-value*	**< 0.0001**	> 0.9999	> 0.9999	N/A	0.0707	N/A	0.2007	N/A
Total	186 (24.6)	5 (0.7)	4 (0.5)	N/A	4 (0.5)	2 (0.3)	3 (0.4)	756

P/T: Positive samples over the total of animals tested. N/A: Non-applicable. *P-*values were calculated with the chi-square and Fisher exact tests between the dry and the rainy seasons.

## Discussion

The aim of this study was to determine the seroprevalence of RVFV in cattle, sheep, and goats in two livestock markets in Yaoundé, Cameroon. The capture and competitive ELISAs used to screen for IgM and IgG targeted the nucleoprotein, which is an immunodominant antigen among phleboviruses and the best candidate for the serodiagnosis of RVFV infections [27–30]. Additionally, the IDvet diagnostic ELISA used in our study has been demonstrated high sensitivity and specificity for the detection of anti-RVFV antibodies [31].

Although it would have been advantageous to conduct the survey in farms, markets offer many interesting features. Indeed, animals from diverse provenances can be assessed with fewer logistical resources. Therefore, evaluating the prevalence of RVFV in markets is a cost-effective way to improve the surveillance of this disease and gain information about its prevalence in different settings, which could have been difficult to reach since the main livestock farming model in Cameroon is nomadism. Most of the animals tested in our study in markets were adult males (91.5%) which are more cost-effective than juveniles or females as the price of animals is important to shepherds and animal owners [32]. This represents a limitation of our study since no juvenile was tested. Indeed, sampling adult animals increase the likelihood of finding IgG-positive individuals as the probability of contact with a virus increases with age which therefore inflates the real IgG seroprevalence in this population [33–36]. Juveniles would have contributed to understand the contemporary epidemiology of RVFV in the specific regions of origin of the herds included [37].

We observed an IgG seroprevalence of 42.2% in cattle, which is very high for a country where no RVFV outbreak has ever been recorded in humans, wild or domestic animals. Our results are comparable to the seroprevalence observed in Mozambique and South Africa where RVFV IgG seroprevalence rates of up to 36.9% and 42.9% were observed, respectively [38,39]. However, lower seroprevalence rates of 13 to 27% have been reported in endemic/epidemic countries like Kenya and Tanzania [40,41]. The apparent absence of outbreaks observed in Cameroon may be due to a lack of active RVFV surveillance which goes unnoticed as is the case for other emerging diseases. However, the molecular analysis of RVFV has described up to 15 genetic lineages with variable pathogenicity [42,43]. Therefore, the absence of outbreak despite the high seroprevalence observed may also be due to the activity of a less virulent viral lineage. More studies are necessary to fully understand the epidemiology of RVFV in Cameroon. Moreover, the seroprevalence observed in our study is also higher than previously reported by Rissmann and colleagues [21]. This discrepancy may be linked to the sampling method, which included animals of all ages and regions with low seroprevalence. It has been shown in other studies that anti-RVFV IgG seroprevalence increases with the age of the hosts [33–36].

Anti-RVFV IgG seroprevalence in sheep (2.7%) and goats (2.4%) was lower than in cattle (χ^2^ = 151, *P*-value < 0.0001). This difference could be explained by the fact that cattle are reared nomadically by shepherds, which increases the probability of virus transmission by vectors from other animal species such as wildlife to cattle, whereas small ruminants are reared around houses and thus have less interaction with other wild animals [44]. It may also be attributed to several factors, including differential vector preference as it has been shown that cattle consistently attract about 3-fold as many mosquitoes as those feeding on sheep and goats [39,45]. Such discrepancy in RVFV seroprevalence between cattle and small ruminants has already been described in Cameroon [21,22] and elsewhere in Africa with a persistent lower anti-RVFV IgG seroprevalence in small ruminants [39,46]. Additionally, high IgG seroprevalences were obtained in cattle from Sudan (52.6%) and Chad (34.7%). Sudan is known for recurrent RVFV outbreaks with the latest recorded in 2019, with more than 1000 human cases and almost 98% of them reported death and abortions among their livestock [47–50]. No outbreak of RVFV has been reported in Chad until now, but the country is bordered by Niger and Sudan, which reported RVFV outbreaks respectively in 2016 and 2007–2008, and 2019 [47–49,51]. The presence of cattle from Sudan and Chad in Cameroon can be a route to the introduction of highly pathogenic RVFV in this country. Indeed, seroprevalence study alone cannot assess the virulence or pathogenicity of the strain active in a location, but virulent lineages of RVFV are active in Sudan [49,52]. Therefore, the presence of animals from these countries in Cameroon raises the concern of transboundary transmission of diseases and, highlights the need for a transboundary One Health strategy to control this disease and other zoonotic diseases in countries importing animals or animal products in Central Africa.

Anti-RVFV IgM antibodies were detected in 5 (1.1%) cattle and 2 (1.4%) sheep, mostly from the North region. The fact that IgM was detected in this region indicates that the virus has been active there in the recent past. The overall low IgM seroprevalence observed in cattle and goats may indicate that, although there is high IgG carriage, RVFV has a low level of contemporary circulation, a situation that could explain why no outbreak has yet been reported. Nevertheless, the movement of livestock during the viremic phase has also been associated with the spread of RVFV from highly affected areas to non-endemic areas [53,54]. The IgM seroprevalence observed in sheep, but not in goats could show that sheep are more susceptible to RVF infection than goats [3,55]. The IgM seroprevalence of 1.1% (5/441) in cattle is similar to those obtained in other African countries such as the Central African Republic (CAR), and the Democratic Republic of Congo (DRC) [35,56]. These results suggest a recent activity of RVFV in Cameroon. This is confirmed by the detection of RVFV RNA by qRT-PCR in three IgM-positive samples from the North region of Cameroon. The failure in amplifying the L segment of phleboviruses on these three samples may be due to the relatively low viral load present in the samples. This was shown by the high Ct values obtained after qRT-PCR (Ct values of 39 and 40). Similar results were obtained in 2014 [21], and in other African countries [57]. The failure in RNA detection by qRT-PCR in the other 4 anti-RVFV IgM positive samples is not unusual. In infected animals, the virus remains in the blood for up to 14 days after the infection, while IgM antibodies persist for three to five months after the infection [58]. Therefore, the detection of IgM antibodies in the absence of the viral RNA shows that the animals have been infected recently but the viraemic phase of the disease was already resolved at the time of inclusion in the study.

The high anti-RVFV IgG seroprevalence obtained during the dry and rainy seasons was statistically significant (X^2^:135.45, *P-*value < 0.0002), and we observed a high anti-RVFV IgG seroprevalence in cattle during the dry season than the rainy season. This could be due to the high number of samples collected during the dry compared to the rainy season. Further studies are needed to elucidate this. Although for small ruminants, we had different patterns, the anti-RVFV IgG and IgM seroprevalences were higher during the rainy than the dry season. This is likely linked to an increased number of mosquitoes during the rainy season that could subsequently increase the transmission of arboviral diseases such as RVFV. In several studies, it has been shown that the seropositivity to RVFV was associated with increased numbers of mosquito vectors such as females *Aedes* spp. [1,58,59]. Lastly, our study provides clear evidence of the circulation of RVFV in livestock in Cameroon. It shows that although the virus seems to be present in the three livestock production foci which are the Adamawa, Far-North, and North regions, the latter is likely a currently active transmission focus. Our study also pinpoints the role of transboundary animal movement/trade in the spread of emerging diseases.

## Conclusion

Our study highlights the circulation of RVFV in livestock across many regions of Cameroon and raises the issue of the transboundary spread of emerging diseases from neighboring countries. Despite, the wide distribution of anti-RVFV IgG antibodies in all the locations, acute infections characterized by the presence of IgM and RVFV RNA detection, were mainly found in animals from the North region of Cameroon. These results emphasize the need for further investigations into this area which seems to be an active focus of RVFV transmission. An interdisciplinary approach should be used for future studies to have a global appreciation of RVFV epidemiology in both wild and domestic animals and humans and also to identify the potential vectors of this virus in Cameroon. It would also be interesting to study the ecological drivers which could impact virus transmission. Additionally, the high seroprevalence observed in the absence of any reported outbreak should trigger more epidemiological studies to assess the incidence in the human population, characterize the local strains and assess the susceptibility of RVFV hosts.
